# Structural insights into tubulin detyrosination by vasohibins-SVBP complex

**DOI:** 10.1038/s41421-019-0133-7

**Published:** 2019-12-31

**Authors:** Xi Liu, Hao Wang, Jinying Zhu, Yongchao Xie, Xin Liang, Zeliang Chen, Yue Feng, Yi Zhang

**Affiliations:** 1 Beijing Advanced Innovation Center for Soft Matter Science and Engineering, Beijing University of Chemical Technology, Beijing, 100029 China; 2 Key Laboratory of Livestock Infectious Diseases in Northeast China, Ministry of Education, College of Aninal Science and Veterinary Medicine, Shenyang Agricultural University, Shenyang, Liaoning 110866 China; 3 Tsinghua-Peking Joint Center for Life Science, School of Life Sciences, Tsinghua University, Beijing, 100084 China; 4 Max-Planck Partner Group, School of Life Sciences, Tsinghua University, Beijing, 100084 China

**Keywords:** X-ray crystallography, Post-translational modifications

Dear Editor,

Microtubules are major elements of the cytoskeleton in eukaryotic cells, essential to a wide variety of cellular functions, including cell shape control, cell division, morphogenesis, motility, and motor protein-based intracellular transport^1,2^. Microtubules are dynamic cylindrical polymers assembled from the conserved α- and β-tubulin heterodimers^3^, which contain globular cores forming the tubular structure and negatively charged C-terminal tails with more variable forms exposed at the microtubule surface. Despite the conservation, cells use multiple α- and β-tubulin isoforms with chemically diverse post-translational modifications (PTMs) to adapt to the specialized functions of microtubules^1^. These PTMs include detyrosination/tyrosination, acetylation/deacetylation, polyglutamylation, and polyglycylation, etc^1,4^. Various tubulin isoforms and abundant PTMs are collectively known as the “tubulin code”, which regulates microtubule properties and its interaction with microtubule-associated protein (MAPs)^3,4^.

Detyrosination/tyrosination is one PTM that involves cyclic removal and reincorporation of the C-terminal tyrosine on most α-tubulin isotypes^5,6^. Tubulin tyrosine ligase (TTL) catalyzes the retyrosination of detyrosinated α-tubulin^7^, while recently vasohibins (VASHs)/SVBP complex were reported as the long-sought tubulin-detyrosinating enzymes, encoding tubulin carboxypeptidase (TCP) activity^8,9^. Two VASHs (VASH1 and VASH2) are found in mammalian genomes, with ~50% sequence identity^10^. VASHs were first identified as secreted angiogenesis regulators^10^, and predicted to harbor a transglutaminase-like protease fold^11^. Small vasohibin binding protein (SVBP), a 66-residue protein, acts as a chaperone-like peptide, which is helpful for vasohibin stability and enhances the tubulin carboxypeptidase activity^8,9^. The identification of VASHs as major tubulin-detyrosinating enzymes establishes the nature of molecules that start the detyrosination-tyrosination cycle, and this helps to extend the understanding of the tubulin PTM in cells. Despite genetic and biochemical studies into the tubulin carboxypeptidase activities of vasohibins/SVBP, the detailed molecular mechanism underlying α-tubulin detyrosination is still unclear due to the lack of structures of vasohibins/SVBP. In this study, we determined the crystal structure of human VASH1-SVBP complex, thereby providing insights into the molecular mechanism of α-tubulin detyrosination by the VASH1-SVBP complex.

Due to the poor solubility of VASH1 when expressed alone, we co-expressed the human SVBP with a C-terminal His tag and VASH1 with a C-terminal Strep tag. After purification tests with different fragments of VASH1, a catalytic core-containing complex VASH1^44–315^-SVBP^1–66^ was purified to homogeneity and we solved the structure of the heterodimer at 2.28 Å resolution by Pt-SAD phasing (Fig. 1a, X-ray statistics in Supplementary Table S1). Importantly, the heterodimer was fully active in the detyrosination activity assays with the tubulin heterodimer or GST (glutathione S-transferase) fusion proteins with C-terminal extensions corresponding to the C-terminal sequence of TUBA1A (^440^VEGEGEEEGEEY^451^) as substrates (Fig. 1b, c). One VASH1-SVBP complex exists in the asymmetric unit. The final refined heterodimer structure contains residues from 60 to 304 of VASH1 and residues 25 to 53 of SVBP. The rest residues of the two proteins are invisible probably due to their intrinsic flexibility.

**Fig. 1:**
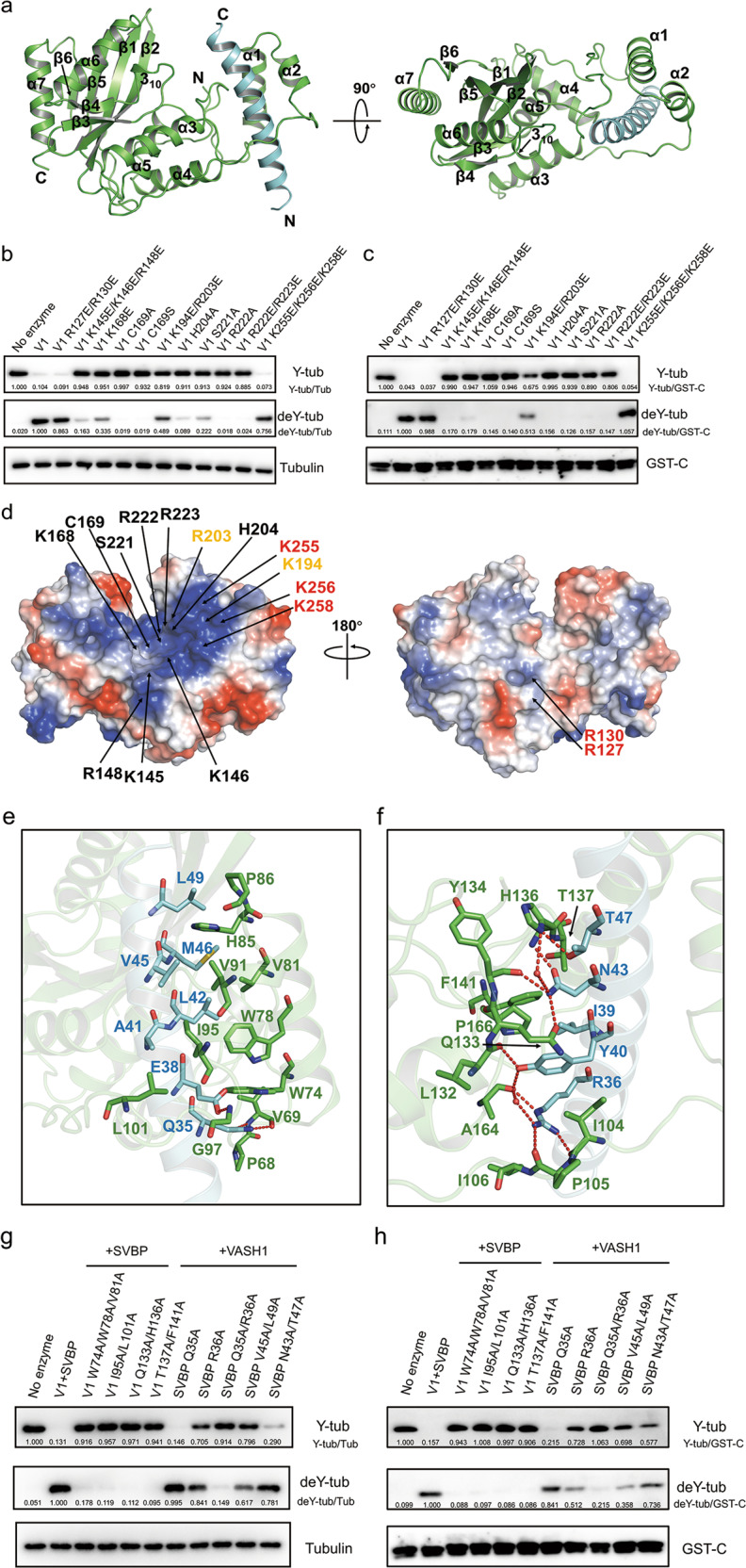
Structure of the VASH1-SVBP complex. **a** Overall structure of the VASH1^44–315^-SVBP^1–66^ heterodimer. VASH1 and SVBP are colored in green and cyan, respectively. Their secondary structures, N- and C-termini are labeled. Two vertical views are shown. **b**, **c** In vitro detyrosination activity assay of wildtype and mutated forms of VASH1-SVBP complex. The enzymes were incubated with purified tubulin dimer (**b**) or α-tubulin tail GST fusion proteins (**c**) for 1 h and 2 h, respectively. **d** Electrostatic surface representation of the VASH1^44–315^-SVBP^1–66^ heterodimer. The residues whose mutation caused markedly decrease, slight decrease and no obvious decrease in activity in (**b**) and (**c**) are colored in black, yellow and red, respectively. **e**, **f** Two interfaces of VASH1-SVBP interaction are shown in cartoon. The residues involved in the interaction are shown as sticks and colored as in (**a**). Hydrogen bonds are shown as red dashed lines and water molecules are shown as red spheres. **g**, **h** In vitro detyrosination activity assay of wildtype and mutated forms of VASH1-SVBP complex. The enzymes were incubated with purified tubulin dimer (**g**) or GST-TUBA1A-C fusion proteins (**h**) for 1 h and 2 h, respectively.

The catalytic core of VASH1 adopts a mixed α/β fold comprising seven α helices, six β strands and a 3_10_ helix (Fig. 1a and Supplementary Fig. S1a). The six-stranded antiparallel β-sheet, flanked by α3/α5 and α6/α7 on the two sides, respectively, constitutes the VASH1 catalytic core. The α1/α2 helical bundle is away from the catalytic core and connected to α3 through an extended loop. In the structure, SVBP forms an extended α helix and is wrapped by VASH1 α1-α5 and the loops among them (Fig. 1a). Vasohibin proteins were previously predicted to be transglutaminase-like cysteine proteases^11^. Consistently, a search in the Dali database returned entries of human coagulation factor XIII (PDB code: 5MHN) and a bacterial protease LapG (PDB code: 4FGO) with the highest Z-scores of 8.0 and 7.9, respectively (Supplementary Fig. S2a, b), both of which exhibit transglutaminase-like folds. Previous bioinformatics study predicted that vasohibin proteins bear a non-canonical Cys169-His204-Ser221 catalytic triad^11^. However, structure analysis revealed that the sidechain nitrogen atom of H204 is actually hydrogen bonded to the carbonyl oxygen atom of L226 (Supplementary Fig. S2c). Therefore, in contrary to the canonical Cys-His-Asp triad of transglutaminase-like cysteine proteases, vasohibin proteins adopt a Cys-His-Leu catalytic triad (Supplementary Fig. S2c). Importantly, C169, H204, and L226 are all conserved among VASH1/2 homologs (Supplementary Fig. S1a). Consistently, the C169A, C169S, and H204A mutations all disrupted the activity of VASH1-SVBP (Fig. 1b, c). The L226A mutation also caused decreased activity of VASH1-SVBP, indicating the sidechain of L226 may also play a role in the activity of VASH1-SVBP (Supplementary Fig. S3).

The VASH1-SVBP electrostatic surface has a conserved, large positively charged region stretching from the active site toward the β1/β2/β5 region and the loop linking α3 and α4 (L34, named similarly hereafter) (Fig. 1d and Supplementary Fig. S1a). This large region should include the one that mediates interaction with the negatively charged α-tubulin tail. In contrary, the dorsal surface lacks such large positively charged region, and thus does not seem to interact with the anionic tubulin tail (Fig. 1d). Due to the failure to obtain the structure of VASH1-SVBP complexed with α-tubulin or its C-terminal tail, we carried out a structure-guided mutagenesis of the conserved surface and active site residues to investigate the substrate binding region in VASH1, using the detyrosination assays with both the purified tubulin heterodimer and α-tubulin tail GST fusion protein as substrates (Fig. 1b, c, Supplementary Figs. S4 and S5). In the active site, mutations of the positively charged residues Lys168, Arg222/Arg223 all disrupted the activity of VASH1-SVBP (Fig. 1b, c). Notably, the mutant of Ser221, which was previously proposed as one of the catalytic triad, also exhibited defective activity. Moreover, mutation of the positively charged residues Lys145/Lys146/Arg148 in the L34 region markedly reduced the activity. For the β1/β2/β5 region, mutations of Lys194/Arg203 and Lys255/256/258 showed no obvious decrease in the activities of VASH1-SVBP, suggesting that this region may not play a major role in the recognition of α-tubulin tail (Fig. 1b, c and Supplementary S1a). Importantly, mutation of the positively charged residues Arg127 and Arg130 to glutamate did not affect the activity of VASH1-SVBP, indicating that this surface is probably not utilized to recognize the α-tubulin (Fig. 1d). Together, we identified that Ser221, the positively charged residues in the active site, the L34 region are important for the detyrosination activity of VASH1-SVBP (Fig. 1d and Supplementary Fig. S4) and these residues are all conserved among the VASH family proteins (Supplementary Fig. S1a). To investigate whether these mutations affect substrate binding, we performed binding assays of the mutants with severely decreased activities by surface plasmon resonance analysis (Supplementary Fig. S6). The results indicated that the K145/K146/R148 region performs an essential role in substrate recognition, but K168, R222, and L226 may play a minor role. Hydrogen bond interactions are formed among Y134, K168, H204, S221, and R222 (Supplementary Fig. S7), suggesting that K168, S221, and R222 may play a role in stabilizing the catalytic center. However, the detailed substrate recognition mechanism still awaits further structural and functional studies into the complex of VASH1-SVBP with tubulin dimer or microtubule.

In the structure, VASH1 and SVBP form extensive interactions, burying a surface area of 2256.5 Å^2^ (Fig. 1a). According to the interface regions of VASH1, the binding interface between VASH1 and SVBP can be roughly divided into two parts (Fig. 1e, f). In the interface involving mainly α1 and α2 of VASH1, extensive hydrophobic interactions are formed between Ala41, Leu42, Val45, and Leu49 of SVBP and Trp74, Trp78, Val81, Val91, Ile95, Leu101 of VASH1 (Fig. 1e). In addition, the carboxyl group of SVBP^E38^ forms a hydrogen bond with the amide group of VASH1^G97^. Moreover, the sidechain of SVBP^Q35^ forms two hydrogen bonds with the main-chain carbonyl oxygen and amide group of VASH1^V69^ (Fig. 1e). In the interface involving the loops linking α2-α5 (L23, L34, and L45), hydrophilic interactions are primarily involved (Fig. 1f). Typically, the sidechain guanidyl of SVBP^R36^ forms three hydrogen bonds with the carbonyl oxygen atoms of Ile104, Pro105, and Ala164 of VASH1. The hydroxyl group of SVBP^Y40^ forms two hydrogen bonds with the carbonyl oxygen atoms of Leu132 and Ala164 of VASH1 (Fig. 1f). The sidechain of SVBP^N43^ forms hydrogen bonds with the sidechain of VASH1^Q133^, the carbonyl oxygen of VASH1^Y134^, and the main-chain amide of VASH1^H136^. Moreover, hydrophobic interactions are also formed between VASH1^F141^ and SVBP^I39^ (Fig. 1f). Importantly, the interacting residues of SVBP and VASH1 are all conserved among the two protein families, respectively (Supplementary Fig. S1). It has been reported that SVBP is required for the stability and full tubulin carboxypeptidase activity of vasohibins^8,9^. Therefore, we speculate that mutations disrupting VASH1-SVBP interactions will decrease the activity of the VASH1-SVBP heterodimer. The detyrosination assay with tubulin dimer as substrates indicated that although the SVBP^Q35A^ and SVBP^R36A^ single mutants showed partially or little decrease in the activity, the Q35A/R36A double mutation in SVBP markedly disrupted the VASH1-SVBP activity (Fig. 1g, h). Moreover, the V45A/L49A mutations of SVBP to break hydrophobic interactions caused a decrease in the activity in both assays. In turn, mutations of the essential interacting residues of VASH1 also reduced the activity of the heterodimer, including W74A/W78A/V81A, I95A/L101A, Q133A/H136A, and T137A/F141A (Fig. 1g, h and Supplementary Fig. S8). Together, the interactions from SVBP could stimulate the activity of VASH1. Although SVBP does not directly interact with the active site residues of VASH1, it might stimulate the activity of VASH1 through other approaches. For example, SVBP interacts with Ala164/Pro166 in the L45 loop adjacent to the active site Cys169 (Fig. 1f), which may help to stabilize α5 where Cys169 resides. However, the detailed function of SVBP also needs structural studies into the complex of VASH1-SVBP with tubulin dimer or microtubule.

In this study, we have provided detailed structural information of VASH1-SVBP. Through structure-guided mutagenesis, we identified that K168, H204, Ser221, R222, and the L34 region are important for the detyrosination activity of VASH1-SVBP (Supplementary Fig. S9). Disrupting the VASH1-SVBP interactions significantly reduced the activity of VASH1, which might be stimulated by the interactions from SVBP through stabilizing the active site of VASH1. During the preparation of our manuscript, structural and functional studies into VASH1-SVBP complex was very recently reported by four studies^12–15^. In their studies, apo or complex structures of VASH1-SVBP with inhibitors or extreme C-terminal mutant peptides of α-tubulin were reported, thus providing insights into recognition of the α-tubulin tail. However, the overall substrate recognition and the function of SVBP still await future structural and functional studies into the complex of VASH1-SVBP with tubulin dimer or microtubule. In all, our study provides important insights into the molecular mechanism of α-tubulin detyrosination by the VASH1-SVBP complex.

The atomic coordinate and structure factors have been deposited in the Protein Data Bank with PDB ID code 6K81.

## Supplementary information

Supplementary Information

## Supplementary information

**Supplementary Information** accompanies the paper at (10.1038/s41421-019-0133-7).
