# Supplementary material for: Structural insights into tubulin detyrosination by vasohibins-SVBP complex
Source: Cell Discov. 2019 Dec 31;5:65. doi: 10.1038/s41421-019-0133-7 (PMC6937246; doi:10.1038/s41421-019-0133-7)
Supplement: Supplementary file 1 — Supplementary Information [file 41421_2019_133_MOESM1_ESM.pdf]

Materials and Methods

Protein expression and purification

The gene encoding human SVBP (residues 1-66) was synthesized by Genscript and cloned into pET22b vector to produce a recombinant protein with a C-terminal His tag. The gene encoding human VASH1 (residues 44-315) was synthesized by Genscript and cloned into a modified pET28a vector to produce a recombinant protein with a C-terminal Strep tag. The above two plasmids were co-transformed into *Escherichia coli* (*E.coli*) Rosetta cells, and induced by 0.2 mM isopropyl-β-D-thiogalactopyranoside (IPTG) when the cell density reached an OD_{600nm} of 1.0. Recombinant protein complex was purified by Ni-affinity column chromatography and Heparin chromatography (Heparin-A: 25 mM Tris pH 8.0, 300 mM NaCl; Heparin-B: 25 mM Tris pH 8.0, 1 M NaCl), and was further subjected to gel filtration chromatography (Superdex-200 column) in buffer containing 10 mM Tris pH 8.0 and 200 mM NaCl. The purified protein was analyzed by SDS–PAGE. The fractions containing the target protein were pooled and concentrated to 20 mg/mL. All the VASH1 and SVBP mutants were generated by two-step PCR and were subcloned, overexpressed and purified in the same way as wild-type protein.

The DNA encoding the C-terminal sequence of TUBA1A (^{440}VEGEGEEEGEEY^{451}) was cloned into pGEX6p-1 vector. The GST fusion protein was expressed as described above. The cells were harvested, re-suspended in lysis buffer (1×PBS, 2 mM DTT and 1 mM PMSF) and lysed by sonication. The cell lysate was centrifuged at 20,000 g for 45 min at 4 ℃ to remove cell debris. The supernatant was applied onto a self-packaged GST-affinity column (2 mL glutathione Sepharose 4B; GE Healthcare) and contaminant proteins were removed with wash buffer (lysis buffer plus 200 mM NaCl). The fusion protein was then eluted by the buffer containing 50 mM Tris pH 7.5, 200 mM NaCl, 50 mM reduced glutathione and 2 mM DTT. The eluant was concentrated and further purified using a Superdex-200 (GE Healthcare) column equilibrated with a buffer containing 10 mM Tris pH 8.0, 200 mM NaCl, and 5 mM DTT. The purified

protein was analyzed by SDS–PAGE. The fractions containing the target protein were pooled, concentrated to 20 mg/mL and stored at -80 ℃.

Crystallization, data collection and structure determination

The VASH1$^{44-315}$-SVBP$^{1-66}$ complex was concentrated to 20 mg/mL in 10 mM Tris-HCl pH 8.0 and 200 mM NaCl. Crystals were grown using the hanging-drop vapor diffusion method. Crystals of VASH1-SVBP complex were grown at 18 ℃ by mixing an equal volume of the protein (20 mg/mL) with reservoir solution containing 0.2 M calcium acetate, 0.1 M Tris pH 7.0 and 20 % PEG 3000. The crystals grew to full size in about two weeks. The crystals were cryoprotected in the reservoir solution containing 10 % glycerol before its transferring to liquid nitrogen. The crystals were soaked into 1 mM $K_2Pt(NO_2)_4$ for 25 hours and flash-frozen in the liquid nitrogen.

All the data were collected at SSRF beamline BL17U1 and BL19U1, integrated and scaled using the HKL2000 package[1]. Further processing was carried out using programs from the CCP4 suite[2]. The initial model was solved by Autosol in PHENIX[3] and refined manually using COOT[4]. The structure was further refined with PHENIX[3] against the native data. The final structure was obtained through several rounds of refinement by PHENIX and COOT.

Purification of recombinant tubulin

The DNA encoding the human α1B tubulin (NP_006073) with an internal His-tag and a PreScission protease-cleavable C-terminally FLAG-tagged βIII tubulin (NP_006077.2) were cloned into pFastBac™-Dual vector as described[5]. The recombinant tubulins were expressed using the Bac-to-Bac system (Invitrogen) in SF9 or HighFive cells. Cells were lysed by sonication in BRB80 buffer (80 mM PIPES pH 6.9, 1 mM $MgCl_2$, 1 mM EGTA) with addition of 0.5 mM ATP, 0.5 mM GTP and 1 mM PMSF. Further, we used anti-flag G1 affinity resin (GenScript) column and Ni-NTA column (Qiagen) for purification. After desalting, the purified tubulin dimer was in BRB80, 20 μM GTP.

In vitro detyrosination assay

In the *in vitro* detyrosination assay, 1.5 μM purified recombinant tubulin dimer or 2 μM GST fusion proteins) in 100 mM MES at pH 6.7, 1 mM EGTA and 1 mM $MgCl_2$ were incubated with 0.6 μM wildtype or mutated forms of the VASH1-SVBP heterodimer. The reactions were incubated for one hour and two hours at 37 ℃, with purified tubulin dimer and GST fusion proteins as substrates, respectively. The enzyme concentration was 0.2 μM in the assays of the SVBP-VASH1 interaction mutants. The gels were blotted on nitrate cellulose strips (Sartorius Stedim # 11327-41BL) in standard blotting buffer containing 20% ethanol. Blocking of the immunoblot was done in 5% milk in TBS-Tween20. Antibodies used for immunoblot analysis: α-tubulin (DM1A; Santa Cruz #32293); detyrosinated α-tubulin (Merck Millipore AB3201); tyrosinated tubulin (Merck Millipore AB3201, ABT171); GST (Jiaxuan Biotech, JX1007).

Surface Plasmon Resonance (SPR) assay

The SPR analysis was performed using a BIAcore T200 machine (GE Healthcare) at room temperature (25 ℃). The recombinant tubulin dimer was immobilized on flow cell 4 of Series S sensor chip CM5 using the standard amine-coupling method (GE Healthcare) to about 400 Response Unit (RU). The flow cell 3 was immobilized blank as a reference. To collect data for kinetic analysis, a concentration series of WT and mutant VASH1/SVBP proteins in binding buffer (20 mM HEPES pH 7.5, 200 mM NaCl, and 0.05 % (v/v) Tween-20) were injected over the chip at a flow rate of 30 μL/min. The complex was allowed to associate for 90 s and dissociate for 420 s. Data was analyzed with Biacore T200 evaluation software by fitting to a 1:1 Langmuir binding fitting model.

Circular Dichroism

Circular dichroism measurements were conducted with Chirascan-plus CD Spectrometer (Applied Photophysics). All the data were collected with 0.5-0.7 mg/mL VASH1/SVBP complex samples in 10 mM Tris pH 8.0, 50 mM NaCl buffer over a wavelength range of 190–260 nm, with 1 nm increments, in a 0.1 mm path length

rectangular cuvette at 25 ℃. All the measurements were performed in triplicate, and the results were averaged.

Supplementary figures

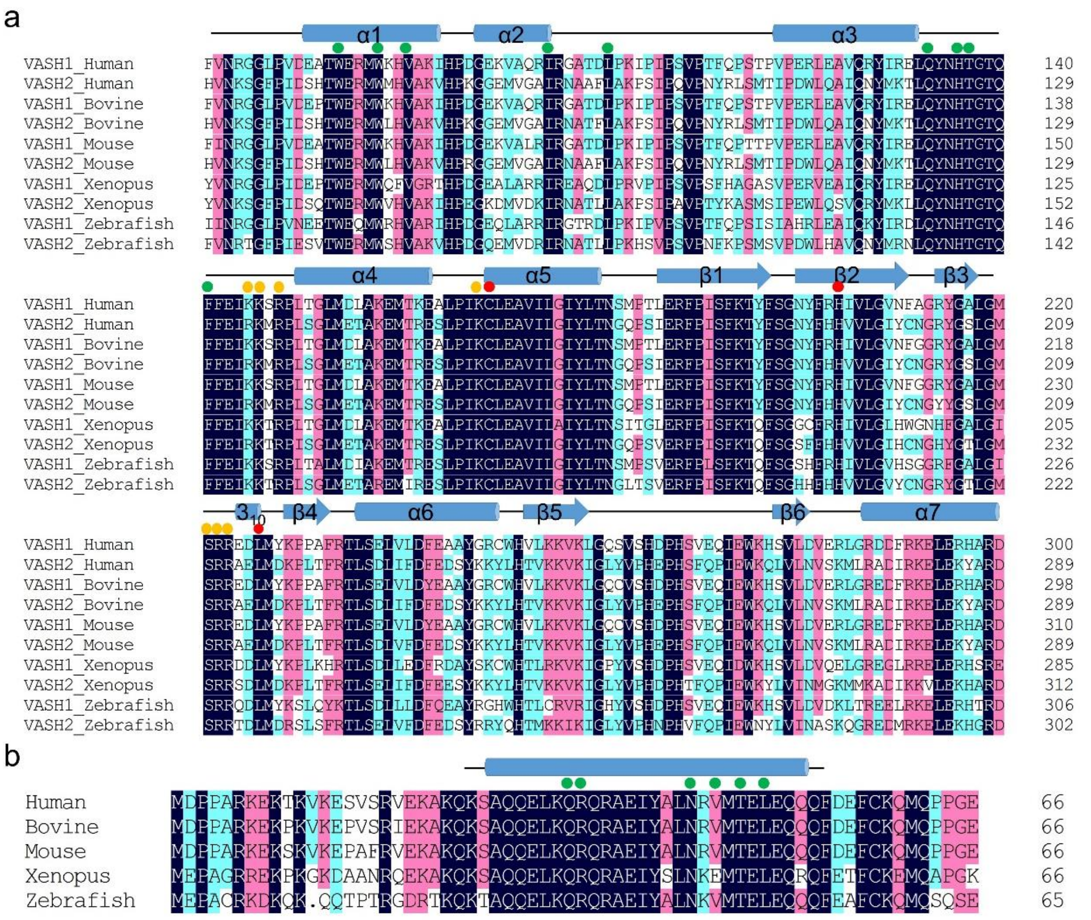

Supplementary Figure S1. Sequence alignment of vasohibin and SVBP family.

(a-b) Sequence alignment of vasohibin family (a) and SVBP family (b) from different species, including *Homo sapiens* (human, UniProt code: Q8N300), *Bos taurus* (Bovine, Q32LJ0), *Mus musculus* (Mouse, Q99LQ4), *Xenopus laevis* (Xenopus, A0A1L8FHH7) and *Danio rerio* (Zebrafish, P0C8M3). The catalytic triad residues, residues important for detyrosination identified by mutagenesis and the residues involved in VASH1-SVBP interaction are marked with red, yellow and green spheres.

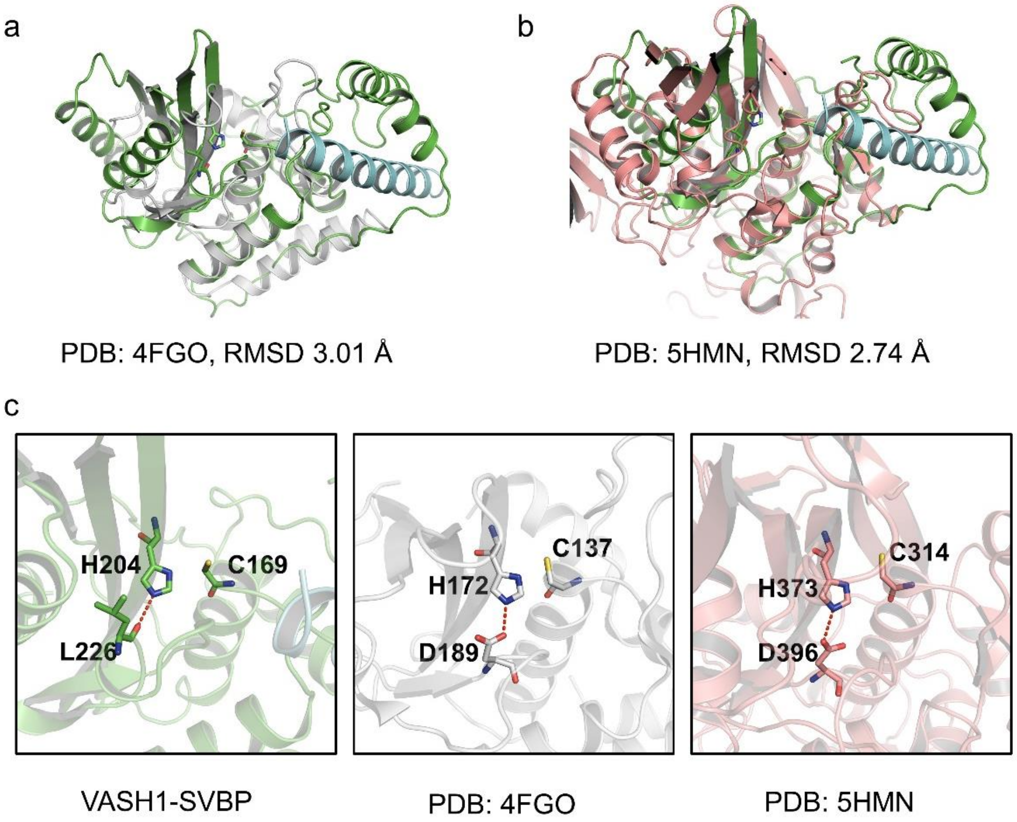

Supplementary Figure S2. Structural alignment of VASH1-SVBP with human coagulation factor XIII and LapG from *Legionella pneumophila*.

(a-b) Structural alignment of VASH1-SVBP (colored as in Fig. 1a) with human coagulation factor XIII (grey, PDB code: 4FGO) and LapG from *Legionella pneumophila* (pink, PDB code: 5HMN).

(c) Active site comparison among the structures in a and b. The three residues of the catalytic triad are shown as sticks and red dashed lines represent hydrogen bonds.

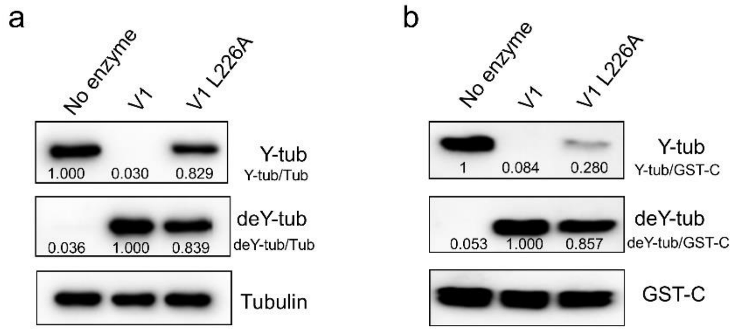

Supplementary Figure S3. *In vitro* detyrosination activity assay of wildtype and VASH1 L226A/SVBP complex.

The enzymes were incubated with recombinant tubulin dimer (a) or α-tubulin tail GST fusion proteins (b) for 1 hour and 2 hours, respectively.

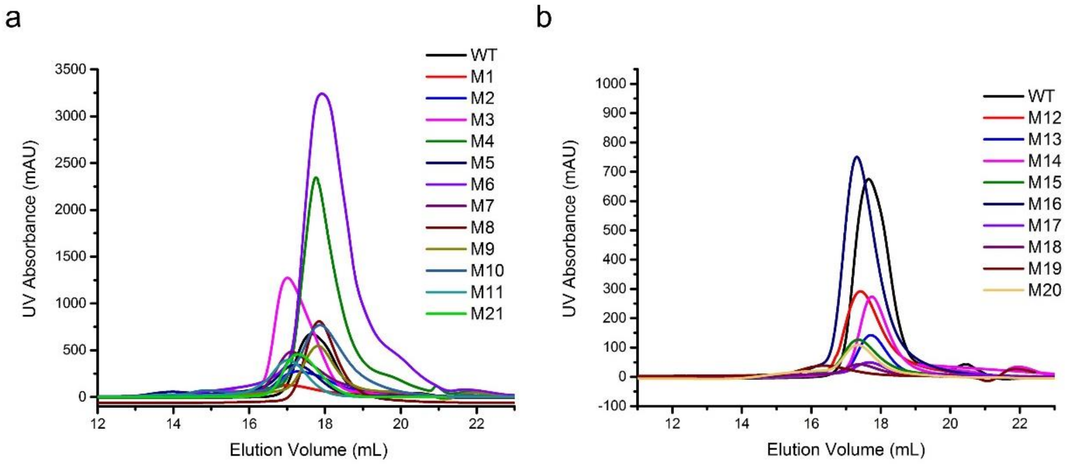

Supplementary Figure S4. Gel filtration profiles of proteins used in this study.

(a) Gel filtration profiles of wildtype and mutants of VASH1-SVBP used in Fig. 1b-c and Supplementary Fig. S3. WT represents wildtype, and M1-M11 and M21 are SVBP complexed with VASH1 mutants: M1 (H204A), M2 (C169A), M3 (C169S), M4 (R127E/R130E), M5 (K145E/K146E/R148E), M6 (K168E), M7 (S221A), M8

(R222A), M9 (R222E/R223E), M10 (K194E/R203E), M11 (K255E/K256E/K258E) and M21 (L226A).

(b) Gel filtration profiles of wildtype and mutants of VASH1-SVBP used in Fig. 1g-h. M12-M16 are VASH1 complexed with SVBP mutants: M12 (Q35A/R36A), M13 (Q35A), M14 (R36A), M15 (V45A/L49A) and M16 (N43A/T47A). M17-M20 are SVBP complexed with VASH1 mutants: M17 (I95A/L101A), M18 (W74/78A/V81A), M19(Q133A/H136A) and M20 (T137A/F141A).

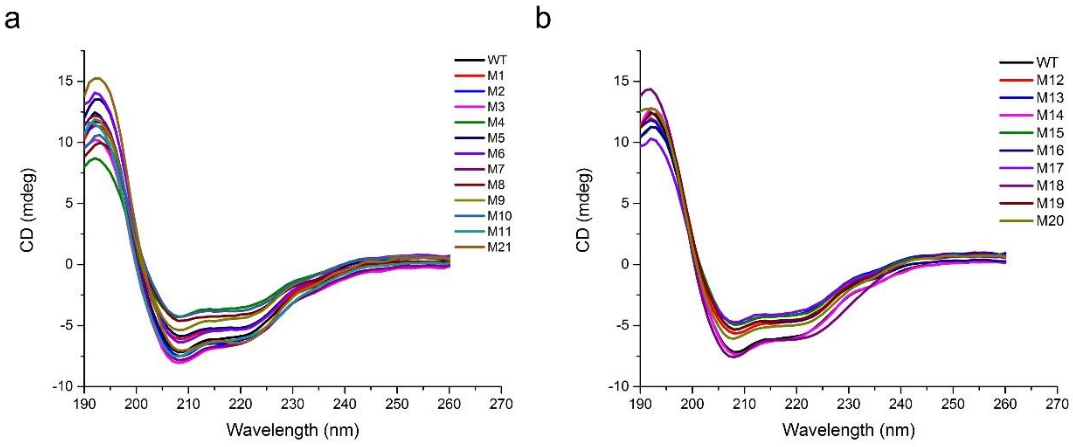

Supplementary Figure S5. Circular dichroism (CD) spectrum of proteins used in this study.

(a) CD spectrum of the protein samples in Supplementary Fig. S4a.

(b) CD spectrum of the protein samples in Supplementary Fig. S4b.

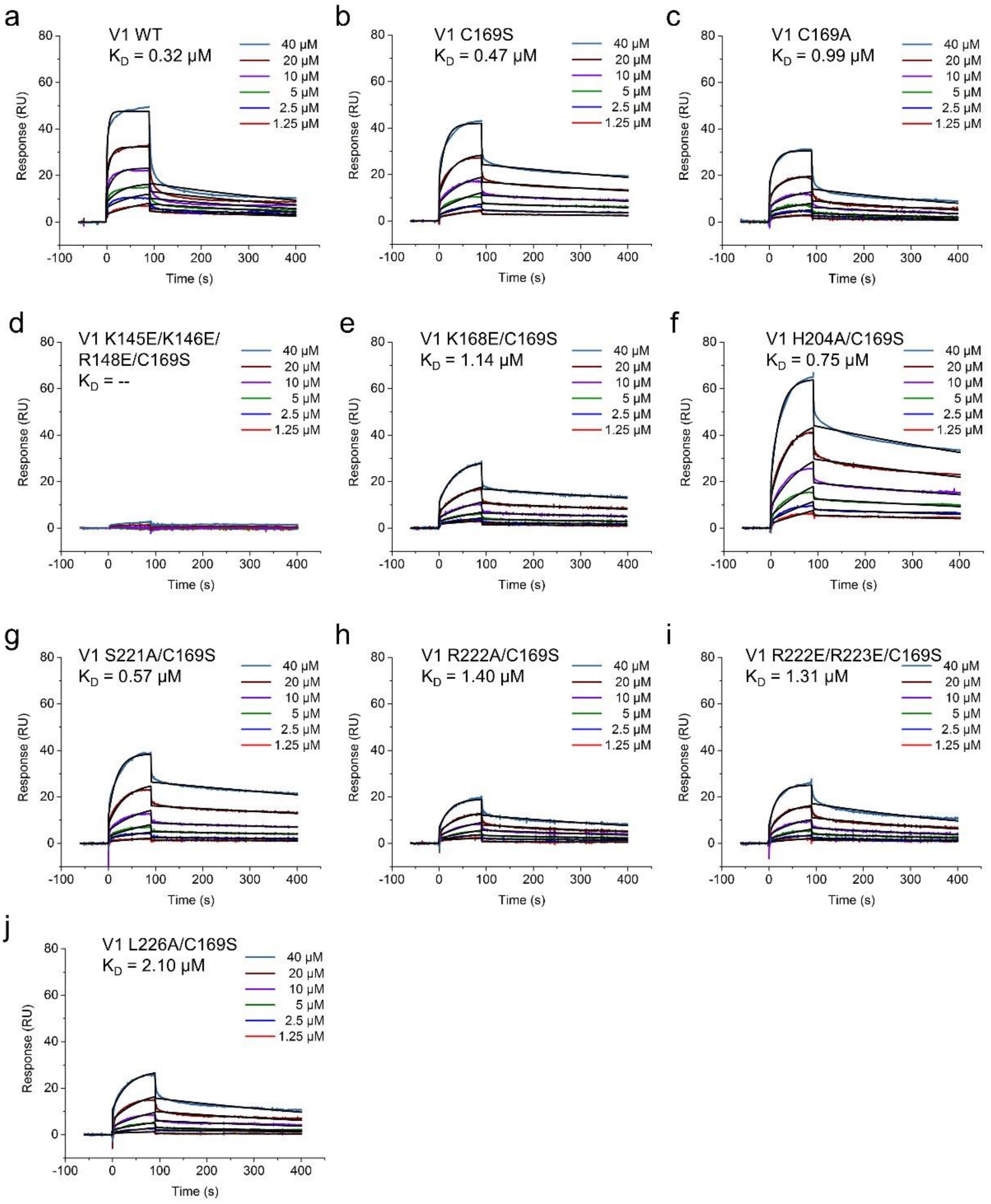

Supplementary Figure S6. Surface Plasmon Resonance (SPR) assay

(a-j) Binding affinities of recombinant tubulin dimer with WT and mutated forms of VASH1/SVBP complex, measured by SPR. SPR curves (colored curves) were fit kinetically using a 1:1 Langmuir binding model (black lines). Data shown are representative of three independent experiments.

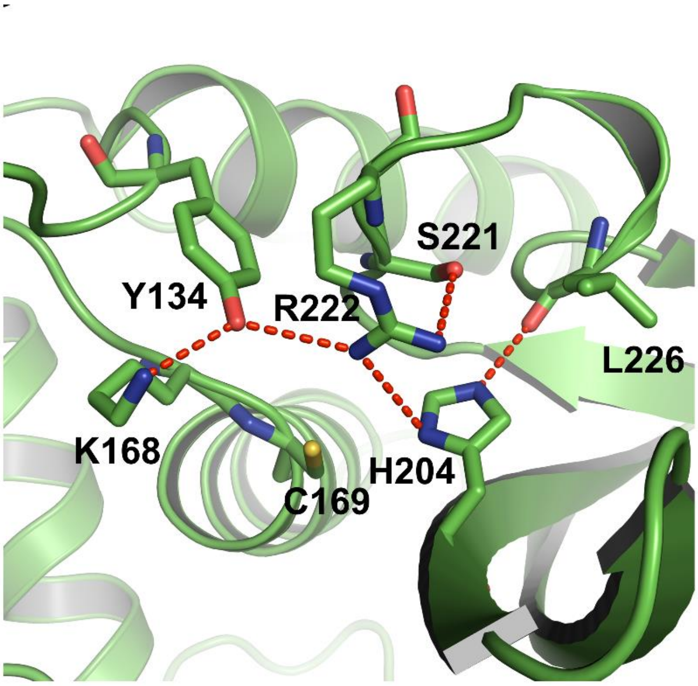

Supplementary Figure S7. Catalytic center of VASH1/SVBP complex

VASH/SVBP complex is shown in cartoon model with some residues in the catalytic center shown in sticks. Hydrogen bonds are shown as red dashed lines.

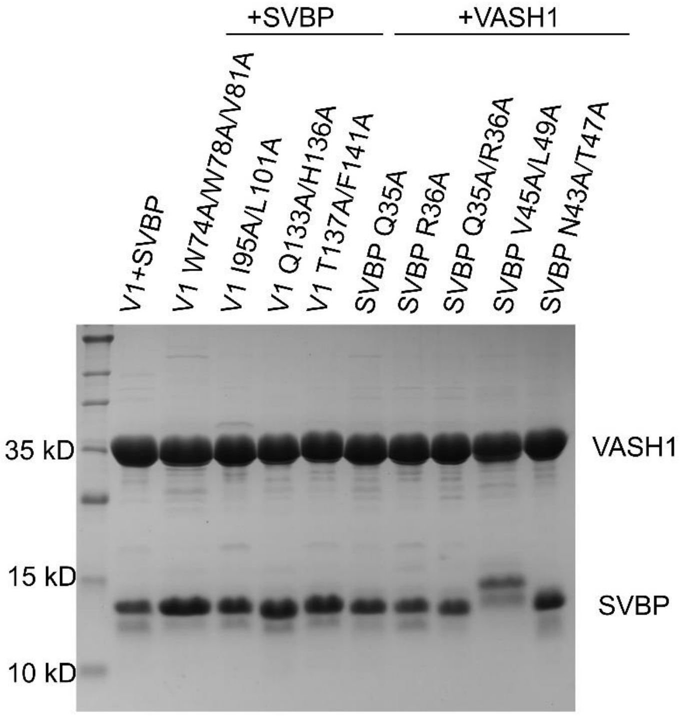

Supplementary Figure S8. SDS-PAGE gel of the samples used in Fig. 1g and 1h

Twelve μg of total protein was loaded into each lane and subjected to SDS-PAGE gel, followed by Coomassie blue staining.

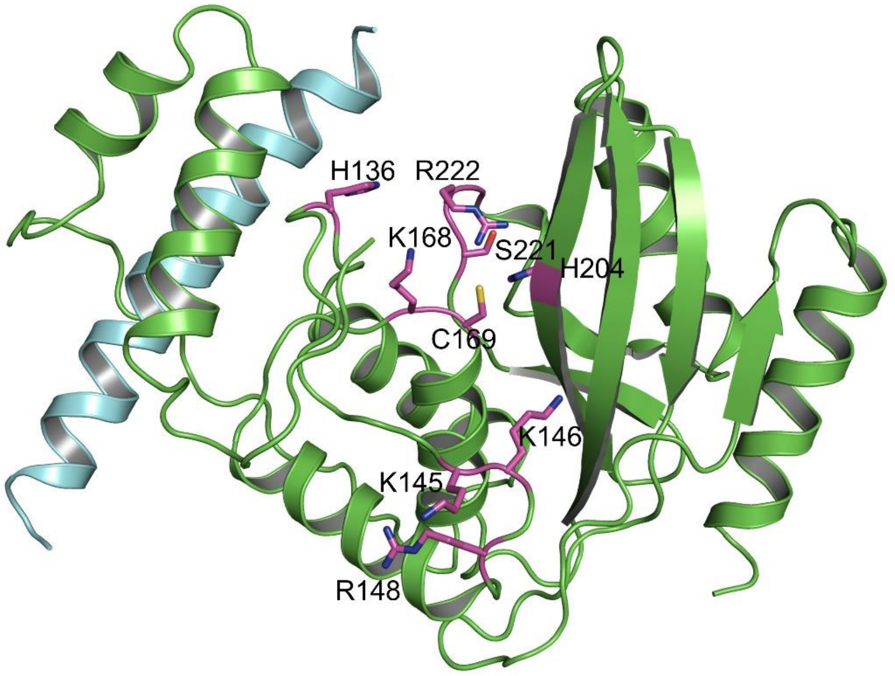

Supplementary Figure S9. Molecular determinants of tubulin detyrosination.

VASH1-SVBP cartoon model with residues important for tubulin detyrosination in stick atomic representation.

Supplementary Table S1 Data collection and refinement statistics

	VASH1-SVBP-Pt-SAD	VASH1-SVBP
**Data collection**		
Space group	P21212	P21212
Cell dimensions		
a, *b*, *c* (Å)	70.76, 127.02, 44.53	70.45, 129.00, 44.54
(°)	90.00, 90.00, 90.00	90.00, 90.00, 90.00
Resolution (Å)	50-2.90 (3.00-2.90)	50-2.28 (2.36-2.28)
R_{sym} or R_{merge} (%)	12.4 (79.9)	9.1 (85.9)
$I/\sigma(I)$	23.5 (1.54)	30.14 (2.78)
Completeness (%)	99.9 (98.9)	99.6 (99.5)
Redundancy	13.1 (9.9)	11.1 (9.4)
**Refinement**		
Resolution (Å)		36.65-2.28 (2.36-2.28)
Unique reflection		18802 (1576)
R_{work} / $R_{free}^{\#}$		0.2043/0.2512
No. atoms		2316
Protein		2239
Ligand/ion		0
Water		77
B factors		36.90
Protein		36.91
Ligand/ion		
Water		36.59
Ramachandran Plot		
favored (%)		97.78
allowed (%)		2.22
outliers (%)		0
R.m.s. deviations		
Bond lengths (Å)		0.006
Bond angles (°)		0.97

For each structure one crystal was used. [a]Values in parentheses are for highest-resolution shell.

$^{\#}R_{free}$ was calculated with 5 % of the reflections selected

References

1. Otwinowski Z, M.W. Processing of X-ray diffraction data collected in oscillation mode. *Macromolecular Crystallography, Pt A*, 307-326 (1997).
2. Winn, M.D. et al. Overview of the CCP4 suite and current developments. *Acta Crystallogr D Biol Crystallogr* **67**, 235-42 (2011).
3. Adams, P.D. et al. PHENIX: building new software for automated crystallographic structure determination. *Acta Crystallogr D Biol Crystallogr* **58**, 1948-54 (2002).
4. Emsley, P. & Cowtan, K. Coot: model-building tools for molecular graphics. *Acta Crystallogr D Biol Crystallogr* **60**, 2126-32 (2004).
5. Vemu, A. et al. Structure and Dynamics of Single-isoform Recombinant Neuronal Human Tubulin. *J Biol Chem* **291**, 12907-15 (2016).
